# Highland Folk Medicine

**Published:** 1922-08

**Authors:** 


### HIGHLAND FOLK MEDICINE.

### SOME STRANGE CUSTOMS.

THE advent of modern customs and the opening up of even the once inaccessible parts of the Highlands by motor vehicles are fast sounding the knell of the "wise women" and "folk-doctors" who formerly played such a part in the life of the simple folk of these remote regions. Nevertheless, there are still a few who practise, and quite a number of the older generation who believe in them. These "doctors" are now, however, rather chary of trying their skill on strangers, and to become a patient it is often necessary to get a mutual friend to effect an introduction, who must at least earnestly profess his belief in his adviser before a cure will be prescribed.

Any particular birth or other body marks play a part in the diagnosis. The "wise woman" (for the majority of these folk-doctors are women) usually engages her patient in a preliminary conversation regarding the weather, crops, fishing, &c., whilst taking stock of any peculiarities he or she may possess. Moles appear to be a clear guide as to the temperament, just as teeth widely set indicate that their owner may be expected to make a safe recovery from illness, while teeth closely set show that the patient is miserly and melancholic, and more difficult to cure.

This preliminary diagnosis, however, seldom decides the exact disease or illness from which the patient is suffering, and recourse is usually had to the following strange way of settling the matter. The "disease" suspected is "named," then a woollen thread is wound round a horn spoon three times, and the ends placed together. The spoon is passed three times round the "crook" (or hook from which pots are suspended in the kitchen). If the thread falls off in the process it is certain that the patient does not suffer from the disease suspected. Another and another is named until the thread remains on, and then it is plain that the last named is that from which the patient is suffering. It is then a case of "apply the usual remedies," many of which, in the writer's opinion, are infinitely worse than the disease. In one case the medicine prescribed was the eating of a cooked mouse, which the poor, unsuspecting patient actually did, mixed up with his food, and is reported to have recovered! Heart disease is regarded by the "folk-doctors" as a serious thing, and the diagnosis for it is special. The patient is told to kneel on the floor near the fire; a sieve is placed on his head, and in the sieve is a large bowl full of water. Into this bowl molten lead is poured through iron—the finger-hole of a scissor blade is often used, and it is held that one of the pieces of lead is sure to assume the rough shape of a heart. If this supposed shape has in it a scallop or hole, then heart disease is a certainty; and the lead is to be melted again and poured into the water, over and over again, until a neatly shaped piece bearing some resemblance to a heart is produced, with no hole, when the patient is considered cured.

When a disease proves to be stubborn and not amenable to treatment, as in cases of general debility which the "wise women" attribute to witchcraft, then three stones are to be taken from underneath a bridge, and marked for head, heart and limbs respectively; these are to be put into the fire till red hot, and then dropped into water, which the patient has to drink in large doses.

Some of the "folk-doctors," known as "skilly women," specialise in the removal of wens. The writer was told of one case, but does not vouch for its authenticity. A young girl suffering from a growth of this kind was treated by one of these experts (?), who prescribed a morning walk over the moors each day to the "doctor's shop." When she reached it, she was told to sit quite still while the woman got ready a needle and knife. The needle was pointed to the wen, and then, acting as if the needle were actually holding the wen fast, she pretended to go through the process of cutting it off, repeating the while, seven times over, a Gaelic incantation which, being translated, runs:—

> "The big mountain, the little mountain;
> The black dog, the spotted dog,
> The brindled dog."

After all this was done, the cure was complete, and the wen is reported to have disappeared.

The cures for warts, which are fairly common in the Highlands, do not sound exactly pleasant, but "faith will remove mountains"! Here are half a dozen popular remedies: Rub them with water found lying on a flat stone in a graveyard; rub them with pig's blood; rub them with eel's blood; spit on them each morning before breakfast; rub them against a snail, then hang the snail by a cord to a tree, and as the snail "dries" the warts disappear; go to the seashore at new moon and wash them in mud and sea water.

Usually the beautiful whiteness and regularity of Highland people's teeth is the subject of admiration among visitors from the South; but for all that they are not immune from toothache and defective molars. In order to save the "rising generation," a child who is known to be cutting its first tooth is given a piece of juniper wood to chew as a preventative; but if this precaution does not avert decay, far better than a visit to a dentist is the obtaining of a "line" from a folk-doctor. Such a line as the one here quoted is worn by the patient under the waistcoat next to the heart. The words on it are: "Peter was sitting on a marble stone weeping. Jesus came by and said: 'What ails ye?' Peter answered and said: 'The toothache.' Jesus answered and said: 'Be ye well from that, Peter, and not ye only, but everyone that believes on me, Peter.' May the Lord Jesus Christ bless His own words, and to Him be praise.—Amen."

The belief in the power of the Evil Eye continues strong in some localities even down to the present day, and if anyone has the misfortune to have come under its influence and a spell has been cast over the victim, illness or disaster can only be averted by carrying out the following directions: Seven smooth stones must be procured from a place " where the living and the dead pass "—that is, from underneath a bridge—and the water carried " dumb " from a well is poured over the stones, the patient has some of it sprinkled on him in the name of the Trinity, and has a quantity given him to drink.

Scrofula, or the King's Evil, is here, as in the Lowlands, believed to be curable by having the seventh son of the same father and mother visiting on seven successive days the patient before partaking of food, washing the afflicted part with water from a well facing the north, and during the process repeating some incantation which neither patient or doctor will divulge, and concluding by spitting in the water. The same seventh son is reputed also to have the power of the " magic foot "—that is, when anything goes wrong with a person's toes he is able to effect a cure by simply standing on them.

All the minor ailments have their own specific cures. Thus, sore eyes are believed by some to be cured by the wearing of earrings, which explains why men may sometimes be seen wearing them. And do not many men and women, even at the present time, wear what they call a galvanic ring for rheumatism, which ought to be quite as effective as carrying a potato in one's pocket for the same purpose—a belief common in certain parts o' England !

### PARAFFIN WAX FOR FLOORS.

**By G. Cadogan Rothery.**

A SPECIAL form of waxing wood flooring is adopted in French and Belgian hospitals and some schools. It is a practical and cheap sanitary method which deserves to be noted.

The method is applicable to parquet or ordinary board flooring, and to new and old wood. The floor is first thoroughly scrubbed, then washed with a warm solution of potash. A mastic is prepared by mixing together 1 part raw sienna, 1 part raw umber, 2 parts finest whiting, and 2 parts liquid fish glue. This makes a stiff, plastic mass, which is used to fill cracks between boards and fill up any depressions, knot holes, &c. Paraffin wax is placed in an iron pot over the fire to melt. It will melt at between 113 deg. to 122 deg. Fahr. if derived from coal-tar, and from 89 deg. to 126 deg. Fahr. if from bituminous shale, and will boil just below 446 deg. Fahr. The paraffin wax must be stirred the whole time and should not more than half fill the pot, while it is well to have at hand a box of sand or dry earth to extinguish flames in case of accidents. The wax should be spread by means of ladles with spouts, as thinly as possible in strips about three feet wide. It soon begins to solidify in the form of a transparent jelly. At this stage it is necessary to remove any superfluous wax by means of a wood plane or a scraper at the end of a pole. The wax must be taken off in thin shavings, while the wax is still warm and soft. It soon hardens and is then difficult to deal with. The surface is rubbed over with iron filings or other suitable fine abrasive, in order to get a perfectly smooth, polished coating. For parquet or new boards one kilogramme of wax will suffice for four square metres. If the wood is old and spongy, rather more will be required.

If well done, this surface will remain in perfect condition for two years. Then a second application, requiring about half the amount of wax, is advisable, and after that a yearly application of less than a quarter of the amount. To keep the flooring in good condition no periodical waxing and elaborate polishing are necessary. All that is needed for cleaning is the passing over the boards a cloth dipped in some antiseptic fluid, and then rapid polishing with a woollen cloth. This form of waxing gives a perfectly impermeable coating, which resists alkalis and acids, even sulphuric acid.

